# 3-(1-Adamant­yl)-6-methyl-3-(3-methyl­benz­yl)isochroman-1-one

**DOI:** 10.1107/S1600536809015888

**Published:** 2009-05-07

**Authors:** Eva Babjaková, Marek Nečas, Robert Vícha

**Affiliations:** a Department of Chemistry, Faculty of Technology, Tomas Bata University in Zlin, Nám. T. G. Masaryka 275, Zlín,762 72, Czech Republic; b Department of Chemistry, Faculty of Science, Masaryk University in Brno, Kamenice 5, Brno-Bohunice, 625 00, Czech Republic

## Abstract

In the title compound, C_28_H_32_O_2_, the oxanone ring adopts distorted half-boat conformation with the following Cremer and Pople puckering parameters: *Q* = 0.619 (2) Å, *θ* = 0.75 (19) and *ϕ* = 172 (13)°. The dihedral angle betwen two benzene rings is 21.32 (7)°. The adamantane unit consists of three fused cyclo­hexane rings in classical chair conformations, with absolute values of C—C—C—C torsion angles in the range 57.5 (2)–60.9 (2)°. Weak inter­actions of the type C—H⋯O link mol­ecules of each enanti­omer into chains parallel to the *b *axis and lying about inversion centers. The crystal packing is also stabilized by inter­molecular π-π stacking inter­actions [centroid–centroid distance of 3.8566 (11) Å].

## Related literature

For related structure and the preparation method, see: Vícha *et al.* (2006 ▶). For the biological activity of related compounds, see: Buntin *et al.* (2008 ▶); Bianchi *et al.* (2004 ▶). For puckering parameters, see: Cremer & Pople (1975 ▶).
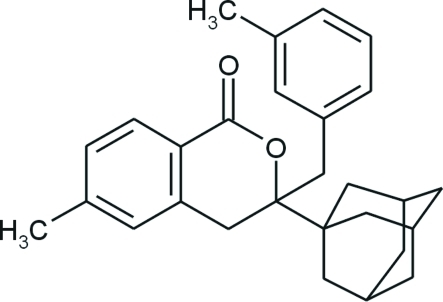


## Experimental

### 

#### Crystal data

C_28_H_32_O_2_
*M* *_r_* = 400.54
Monoclinic, 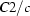

*a* = 25.691 (5) Å
*b* = 6.8474 (14) Å
*c* = 24.465 (5) Å
β = 95.62 (3)°
*V* = 4283.1 (15) Å^3^
*Z* = 8
Mo *K*α radiation
μ = 0.08 mm^−1^
*T* = 120 K
0.50 × 0.40 × 0.40 mm

#### Data collection

Kuma KM-4-CCD diffractometer
Absorption correction: multi-scan (**CrysAlis RED**; Oxford Diffraction, 2006 ▶) *T*_min_ = 0.928, *T*_max_ = 0.976
24394 measured reflections
3768 independent reflections
2791 reflections with *I* > 2σ(*I*)
*R*_int_ = 0.025

#### Refinement

*R*[*F* ^2^ > 2σ(*F* ^2^)] = 0.048
*wR*(*F* ^2^) = 0.151
*S* = 1.09
3768 reflections
273 parameters
H-atom parameters constrained
Δρ_max_ = 0.74 e Å^−3^
Δρ_min_ = −0.30 e Å^−3^

### 

Data collection: *CrysAlis CCD* (Oxford Diffraction, 2006 ▶); cell refinement: *CrysAlis RED* (Oxford Diffraction, 2006 ▶); data reduction: *CrysAlis RED*; program(s) used to solve structure: *SHELXS97* (Sheldrick, 2008 ▶); program(s) used to refine structure: *SHELXL97* (Sheldrick, 2008 ▶); molecular graphics: *ORTEP-3* (Farrugia, 1997 ▶) and *Mercury* (Macrae *et al.*, 2008 ▶); software used to prepare material for publication: *SHELXL97*.

## Supplementary Material

Crystal structure: contains datablocks global, I. DOI: 10.1107/S1600536809015888/pv2152sup1.cif

Structure factors: contains datablocks I. DOI: 10.1107/S1600536809015888/pv2152Isup2.hkl

Additional supplementary materials:  crystallographic information; 3D view; checkCIF report

## Figures and Tables

**Table 1:** Hydrogen-bond geometry (Å, °)

*D*—H⋯*A*	*D*—H	H⋯*A*	*D*⋯*A*	*D*—H⋯*A*
C23—H23*A*⋯O2^i^	0.95	2.65	3.548 (3)	157
C12—H12*B*⋯O2^i^	0.99	2.28	3.206 (2)	156

Symmetry code: (i) 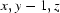.

**Fig. 1.**
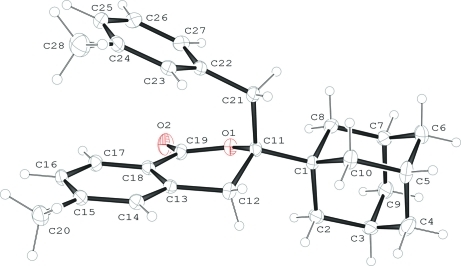
ORTEP (Farrugia, 1997) of the asymmetric unit with atoms represented as 50% probability ellipsoids and H atoms shown as small spheres at arbitrary radii.

**Fig. 2.**
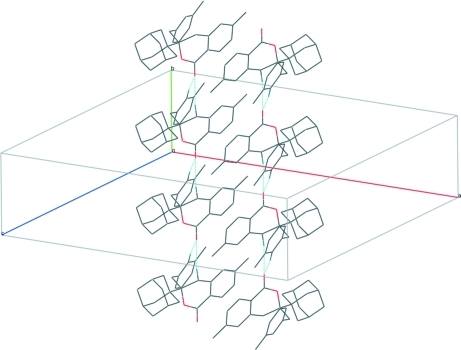
The crystal packing of the title compound showing chains paralel to the b-axis linked via C—H···O weak interactions (dotted lines). H-atoms have been omitted to enhance clarity (except those which are involved in H-bonding).

## supplementary crystallographic information

### Comment

The title molecule is related to the isochromanone derivatives that are generally known as regulators of plant growth (Bianchi *et al.*, 2004). In the dependence on their chemical structure and concentration they can act either as inhibitors or stimulators in these processes. Some substituted isochromanones isolated from myxobacteria strains were introduced as antifungal agents (Buntin *et al.*, 2008).

The structure of the title compound (Fig. 1) consists of two essentially planar benzene rings with the maximum deviations from the best planes of 0.0046 (18) Å for atom C18 (benzene ring C13-C18) and 0.0115 (2) Å for atom C22 (benzyl group). The carbonyl plane (C18/C19/O1/O2) is also planar with the maximum deviation being 0.0073 (2) Å for atom C19. The dihedral angles between two benzene rings and between carbonyl plane and adjacent benzene ring are 21.32 (7) and 12.56 (6)°, respectively. The oxanone ring adopts distorted half-boat conformation with the torsion angles C12–C13–C18–C19, O1–C19–C18–C13 and C11–C12–C13–C18 being -2.3 (3), -10.8 (3) and 32.4 (2)°, respectively. The puckering parameters (Cremer & Pople, 1975) for the oxanone ring are *Q* = 0.619 (2) Å, *θ* = 0.75 (19)° and *φ* = 172 (13)°. The torsion angles describing alignment of adamantane cage and benzyl group C12–C11–C1–C2 and C12–C11–C21–C22 are 50.8 (2) and -69.7 (2)°, respectively. The adamantane cage consists of three fused cyclohexane rings in classical chair conformation, with absolute values of C–C–C–C torsion angles within the range 57.5 (2)-60.9 (2)°. The molecules of each enantiomer are linked *via* C23–H23A···O2 and C12–H12B···O2 weak interactions into chains parallel to the *b*-axis and lying about inversion centers (Table 1 and Fig. 2). The packing of the crystal is stabilized by intermolecular π-π stacking of isochromanone rings with the centroid-to-centroid (*Cg*: C13–C18) distance being 3.8566 (11) Å.

### Experimental

The title compound was prepared by the reaction of 3-methylbenzylmagnesium chloride with adamantane-1-carbonyl chloride in diethyl ether (Vícha *et al.*, 2006). A solution of 3-methylbenzylmagnesium chloride (5 ml, 0.030 mol) in diethyl ether was added to a well stirred solution of adamantane-1-carbonyl chloride (0.8858 g, 0.004 mol) in 5 ml of dry diethyl ether at 273 K. The mixture was stirred for 72 h at room temperature and the reaction mixture was quenched with 15 ml of HCl (1 *M*). After additional 15 min of vigorous stirring, the aqueous layer was separated and washed three times with 15 ml of diethyl ether. Combined organic layers were washed with K_2_CO_3_ (1.16 *M*), dried over Na_2_SO_4_ and evaporated in vacuum. Crude product was purified on column (silica gel; petroleum ether/ethyl acetate, *v*/*v*, 16/1). The title compound was obtained as a colorless crystalline powder (350 mg, 41%), melting point 447–448 K). Crystals suitable for X-ray analysis were acquired by spontaneous evaporation from deuterochloroform at 298 K.

### Refinement

Hydrogen atoms were positioned geometrically and refined as riding using standard *SHELXTL* (Sheldrick, 2008) facilities, with their *U*_iso_ set to either 1.2U_eq_ or 1.5U_eq_ (methyl) of their parent atoms.

### Crystal data

**Table:**

C_28_H_32_O_2_	*F*(000) = 1728
*M**_r_* = 400.54	*D*_x_ = 1.242 Mg m^−^^3^
Monoclinic, *C*2/*c*	Melting point: 448(1) K
Hall symbol: -C 2yc	Mo *K*α radiation, λ = 0.71073 Å
*a* = 25.691 (5) Å	Cell parameters from 3768 reflections
*b* = 6.8474 (14) Å	θ = 3.1–25.0°
*c* = 24.465 (5) Å	µ = 0.08 mm^−^^1^
β = 95.62 (3)°	*T* = 120 K
*V* = 4283.1 (15) Å^3^	Block, colourless
*Z* = 8	0.50 × 0.40 × 0.40 mm

### Data collection

**Table:**

Kuma KM-4-CCD diffractometer	3768 independent reflections
Radiation source: fine-focus sealed tube	2791 reflections with *I* > 2σ(*I*)
graphite	*R*_int_ = 0.025
Detector resolution: 0.06 pixels mm^-1^	θ_max_ = 25.0°, θ_min_ = 3.1°
ω scans	*h* = −26→30
Absorption correction: multi-scan (*CrysAlis RED*; Oxford Diffraction, 2006)	*k* = −8→8
*T*_min_ = 0.928, *T*_max_ = 0.976	*l* = −29→29
24394 measured reflections	

### Refinement

**Table:**

Refinement on *F*^2^	Primary atom site location: structure-invariant direct methods
Least-squares matrix: full	Secondary atom site location: difference Fourier map
*R*[*F*^2^ > 2σ(*F*^2^)] = 0.048	Hydrogen site location: inferred from neighbouring sites
*wR*(*F*^2^) = 0.151	H-atom parameters constrained
*S* = 1.09	*w* = 1/[σ^2^(*F*_o_^2^) + (0.0809*P*)^2^ + 3.6011*P*] where *P* = (*F*_o_^2^ + 2*F*_c_^2^)/3
3768 reflections	(Δ/σ)_max_ < 0.001
273 parameters	Δρ_max_ = 0.74 e Å^−^^3^
0 restraints	Δρ_min_ = −0.30 e Å^−^^3^

### Special details

**Table:**

Geometry. All e.s.d.'s (except the e.s.d. in the dihedral angle between two l.s. planes) are estimated using the full covariance matrix. The cell e.s.d.'s are taken into account individually in the estimation of e.s.d.'s in distances, angles and torsion angles; correlations between e.s.d.'s in cell parameters are only used when they are defined by crystal symmetry. An approximate (isotropic) treatment of cell e.s.d.'s is used for estimating e.s.d.'s involving l.s. planes.
Refinement. Refinement of *F*^2^ against ALL reflections. The weighted *R*-factor *wR* and goodness of fit *S* are based on *F*^2^, conventional *R*-factors *R* are based on *F*, with *F* set to zero for negative *F*^2^. The threshold expression of *F*^2^ > σ(*F*^2^) is used only for calculating *R*-factors(gt) *etc*. and is not relevant to the choice of reflections for refinement. *R*-factors based on *F*^2^ are statistically about twice as large as those based on *F*, and *R*- factors based on ALL data will be even larger.

### Fractional atomic coordinates and isotropic or equivalent isotropic displacement parameters (Å^2^)

**Table:**

	*x*	*y*	*z*	*U*_iso_*/*U*_eq_	
O1	0.12111 (5)	0.58024 (19)	0.42951 (5)	0.0274 (3)	
O2	0.10665 (6)	0.8319 (2)	0.48112 (6)	0.0416 (4)	
C1	0.12676 (7)	0.3506 (3)	0.35689 (7)	0.0249 (4)	
C2	0.07450 (8)	0.4117 (4)	0.32486 (8)	0.0357 (5)	
H2A	0.0461	0.3273	0.3359	0.043*	
H2B	0.0663	0.5482	0.3342	0.043*	
C3	0.07703 (8)	0.3948 (4)	0.26254 (8)	0.0415 (6)	
H3A	0.0426	0.4347	0.2431	0.050*	
C4	0.08847 (10)	0.1837 (4)	0.24825 (9)	0.0508 (7)	
H4A	0.0899	0.1707	0.2081	0.061*	
H4B	0.0603	0.0978	0.2592	0.061*	
C5	0.14076 (10)	0.1229 (3)	0.27842 (9)	0.0424 (6)	
H5A	0.1484	−0.0153	0.2687	0.051*	
C6	0.18356 (9)	0.2548 (4)	0.26050 (9)	0.0406 (6)	
H6A	0.1852	0.2425	0.2204	0.049*	
H6B	0.2178	0.2149	0.2792	0.049*	
C7	0.17210 (8)	0.4660 (3)	0.27493 (8)	0.0337 (5)	
H7A	0.2005	0.5523	0.2634	0.040*	
C8	0.16969 (8)	0.4845 (3)	0.33713 (8)	0.0299 (5)	
H8A	0.1623	0.6219	0.3464	0.036*	
H8B	0.2040	0.4486	0.3565	0.036*	
C9	0.11992 (9)	0.5274 (4)	0.24478 (8)	0.0395 (5)	
H9A	0.1123	0.6649	0.2536	0.047*	
H9B	0.1215	0.5168	0.2046	0.047*	
C10	0.13864 (9)	0.1386 (3)	0.34079 (8)	0.0351 (5)	
H10A	0.1726	0.0972	0.3600	0.042*	
H10B	0.1111	0.0506	0.3523	0.042*	
C11	0.12507 (7)	0.3690 (3)	0.42072 (7)	0.0245 (4)	
C12	0.07750 (8)	0.2678 (3)	0.44128 (7)	0.0255 (4)	
H12A	0.0463	0.2945	0.4152	0.031*	
H12B	0.0834	0.1249	0.4419	0.031*	
C13	0.06683 (7)	0.3338 (3)	0.49754 (7)	0.0240 (4)	
C14	0.04511 (7)	0.2122 (3)	0.53459 (7)	0.0265 (4)	
H14A	0.0368	0.0812	0.5243	0.032*	
C15	0.03512 (7)	0.2773 (3)	0.58642 (7)	0.0286 (5)	
C16	0.04774 (8)	0.4696 (3)	0.60077 (8)	0.0302 (5)	
H16A	0.0414	0.5165	0.6361	0.036*	
C17	0.06931 (8)	0.5930 (3)	0.56449 (8)	0.0294 (5)	
H17A	0.0778	0.7238	0.5749	0.035*	
C18	0.07859 (7)	0.5254 (3)	0.51267 (7)	0.0247 (4)	
C19	0.10280 (8)	0.6571 (3)	0.47482 (8)	0.0276 (5)	
C20	0.01153 (9)	0.1436 (3)	0.62600 (8)	0.0383 (5)	
H20A	0.0237	0.0098	0.6209	0.057*	
H20B	−0.0267	0.1480	0.6193	0.057*	
H20C	0.0222	0.1858	0.6637	0.057*	
C21	0.17657 (8)	0.2921 (3)	0.45125 (8)	0.0326 (5)	
H21A	0.1763	0.1479	0.4485	0.039*	
H21B	0.2059	0.3397	0.4314	0.039*	
C22	0.18831 (8)	0.3455 (3)	0.51107 (8)	0.0298 (5)	
C23	0.17789 (8)	0.2147 (3)	0.55169 (8)	0.0313 (5)	
H23A	0.1640	0.0900	0.5415	0.038*	
C24	0.18731 (8)	0.2613 (3)	0.60726 (8)	0.0353 (5)	
C25	0.20832 (8)	0.4433 (4)	0.62145 (8)	0.0389 (5)	
H25A	0.2145	0.4785	0.6591	0.047*	
C26	0.22031 (8)	0.5736 (4)	0.58156 (9)	0.0392 (5)	
H26A	0.2354	0.6965	0.5918	0.047*	
C27	0.21027 (8)	0.5250 (3)	0.52660 (8)	0.0353 (5)	
H27A	0.2185	0.6153	0.4992	0.042*	
C28	0.17439 (10)	0.1205 (4)	0.64983 (10)	0.0515 (7)	
H28A	0.1786	−0.0129	0.6365	0.077*	
H28B	0.1381	0.1403	0.6578	0.077*	
H28C	0.1979	0.1410	0.6834	0.077*	

### Atomic displacement parameters (Å^2^)

**Table:**

	*U*^11^	*U*^22^	*U*^33^	*U*^12^	*U*^13^	*U*^23^
O1	0.0340 (8)	0.0251 (7)	0.0238 (7)	0.0011 (6)	0.0060 (6)	−0.0008 (5)
O2	0.0616 (11)	0.0269 (9)	0.0376 (9)	0.0005 (7)	0.0125 (7)	−0.0030 (6)
C1	0.0240 (10)	0.0316 (11)	0.0193 (9)	0.0020 (8)	0.0032 (7)	−0.0006 (8)
C2	0.0255 (11)	0.0581 (15)	0.0236 (10)	0.0035 (10)	0.0027 (8)	0.0044 (9)
C3	0.0251 (12)	0.0774 (17)	0.0216 (10)	−0.0017 (11)	0.0003 (8)	0.0049 (10)
C4	0.0537 (16)	0.0751 (18)	0.0243 (11)	−0.0294 (14)	0.0074 (10)	−0.0101 (11)
C5	0.0597 (16)	0.0416 (13)	0.0274 (11)	−0.0029 (11)	0.0122 (10)	−0.0102 (9)
C6	0.0399 (13)	0.0588 (15)	0.0246 (10)	0.0040 (11)	0.0101 (9)	−0.0027 (10)
C7	0.0276 (11)	0.0465 (13)	0.0278 (10)	−0.0056 (9)	0.0062 (8)	0.0019 (9)
C8	0.0280 (11)	0.0364 (12)	0.0252 (10)	−0.0040 (9)	0.0020 (8)	0.0001 (8)
C9	0.0425 (14)	0.0534 (14)	0.0225 (10)	0.0030 (11)	0.0033 (9)	0.0047 (9)
C10	0.0432 (13)	0.0369 (12)	0.0259 (10)	−0.0020 (10)	0.0077 (9)	−0.0039 (9)
C11	0.0276 (11)	0.0243 (10)	0.0219 (10)	0.0040 (8)	0.0039 (8)	−0.0024 (7)
C12	0.0315 (11)	0.0231 (10)	0.0222 (9)	0.0002 (8)	0.0039 (8)	−0.0001 (7)
C13	0.0203 (10)	0.0281 (10)	0.0231 (9)	0.0063 (8)	0.0004 (7)	−0.0012 (8)
C14	0.0252 (11)	0.0285 (10)	0.0259 (10)	0.0014 (8)	0.0027 (8)	−0.0001 (8)
C15	0.0227 (11)	0.0398 (12)	0.0234 (10)	0.0039 (8)	0.0035 (8)	0.0025 (8)
C16	0.0266 (11)	0.0427 (12)	0.0218 (9)	0.0082 (9)	0.0053 (8)	−0.0026 (8)
C17	0.0312 (11)	0.0291 (11)	0.0280 (10)	0.0050 (8)	0.0038 (8)	−0.0047 (8)
C18	0.0227 (10)	0.0273 (10)	0.0240 (9)	0.0068 (8)	0.0024 (8)	0.0012 (8)
C19	0.0326 (12)	0.0259 (11)	0.0243 (10)	0.0052 (8)	0.0025 (8)	−0.0028 (8)
C20	0.0385 (13)	0.0475 (14)	0.0303 (11)	0.0021 (10)	0.0101 (9)	0.0037 (9)
C21	0.0308 (12)	0.0465 (13)	0.0202 (10)	0.0108 (9)	0.0009 (8)	−0.0025 (9)
C22	0.0234 (11)	0.0434 (12)	0.0224 (10)	0.0099 (9)	0.0018 (8)	0.0003 (9)
C23	0.0235 (11)	0.0400 (12)	0.0302 (11)	0.0094 (9)	0.0013 (8)	−0.0025 (9)
C24	0.0272 (11)	0.0541 (14)	0.0254 (10)	0.0145 (10)	0.0064 (8)	0.0055 (9)
C25	0.0307 (12)	0.0591 (15)	0.0262 (11)	0.0079 (10)	−0.0007 (9)	−0.0088 (10)
C26	0.0296 (12)	0.0514 (14)	0.0357 (12)	−0.0026 (10)	−0.0019 (9)	−0.0097 (10)
C27	0.0279 (12)	0.0465 (13)	0.0313 (11)	−0.0013 (9)	0.0012 (9)	0.0000 (9)
C28	0.0539 (16)	0.0593 (16)	0.0423 (14)	0.0055 (13)	0.0102 (11)	0.0120 (12)

### Geometric parameters (Å, °)

**Table:**

O1—C19	1.353 (2)	C12—H12A	0.9900
O1—C11	1.467 (2)	C12—H12B	0.9900
O2—C19	1.209 (2)	C13—C14	1.388 (3)
C1—C10	1.542 (3)	C13—C18	1.388 (3)
C1—C2	1.544 (3)	C14—C15	1.391 (3)
C1—C8	1.548 (3)	C14—H14A	0.9500
C1—C11	1.572 (2)	C15—C16	1.393 (3)
C2—C3	1.537 (3)	C15—C20	1.503 (3)
C2—H2A	0.9900	C16—C17	1.381 (3)
C2—H2B	0.9900	C16—H16A	0.9500
C3—C4	1.523 (4)	C17—C18	1.392 (3)
C3—C9	1.524 (3)	C17—H17A	0.9500
C3—H3A	1.0000	C18—C19	1.474 (3)
C4—C5	1.526 (4)	C20—H20A	0.9800
C4—H4A	0.9900	C20—H20B	0.9800
C4—H4B	0.9900	C20—H20C	0.9800
C5—C6	1.520 (3)	C21—C22	1.510 (3)
C5—C10	1.536 (3)	C21—H21A	0.9900
C5—H5A	1.0000	C21—H21B	0.9900
C6—C7	1.525 (3)	C22—C23	1.383 (3)
C6—H6A	0.9900	C22—C27	1.390 (3)
C6—H6B	0.9900	C23—C24	1.394 (3)
C7—C9	1.524 (3)	C23—H23A	0.9500
C7—C8	1.534 (3)	C24—C25	1.389 (3)
C7—H7A	1.0000	C24—C28	1.481 (3)
C8—H8A	0.9900	C25—C26	1.379 (3)
C8—H8B	0.9900	C25—H25A	0.9500
C9—H9A	0.9900	C26—C27	1.385 (3)
C9—H9B	0.9900	C26—H26A	0.9500
C10—H10A	0.9900	C27—H27A	0.9500
C10—H10B	0.9900	C28—H28A	0.9800
C11—C12	1.532 (3)	C28—H28B	0.9800
C11—C21	1.547 (3)	C28—H28C	0.9800
C12—C13	1.500 (2)		
			
C19—O1—C11	122.58 (14)	C12—C11—C1	112.97 (15)
C10—C1—C2	108.02 (16)	C21—C11—C1	110.29 (15)
C10—C1—C8	108.25 (16)	C13—C12—C11	112.85 (15)
C2—C1—C8	106.96 (16)	C13—C12—H12A	109.0
C10—C1—C11	110.79 (15)	C11—C12—H12A	109.0
C2—C1—C11	112.03 (15)	C13—C12—H12B	109.0
C8—C1—C11	110.63 (15)	C11—C12—H12B	109.0
C3—C2—C1	111.34 (17)	H12A—C12—H12B	107.8
C3—C2—H2A	109.4	C14—C13—C18	118.94 (17)
C1—C2—H2A	109.4	C14—C13—C12	122.69 (17)
C3—C2—H2B	109.4	C18—C13—C12	118.37 (17)
C1—C2—H2B	109.4	C13—C14—C15	121.66 (18)
H2A—C2—H2B	108.0	C13—C14—H14A	119.2
C4—C3—C9	109.82 (19)	C15—C14—H14A	119.2
C4—C3—C2	109.08 (19)	C14—C15—C16	118.26 (18)
C9—C3—C2	109.89 (18)	C14—C15—C20	120.94 (19)
C4—C3—H3A	109.3	C16—C15—C20	120.80 (18)
C9—C3—H3A	109.3	C17—C16—C15	120.99 (18)
C2—C3—H3A	109.3	C17—C16—H16A	119.5
C3—C4—C5	109.19 (18)	C15—C16—H16A	119.5
C3—C4—H4A	109.8	C16—C17—C18	119.81 (18)
C5—C4—H4A	109.8	C16—C17—H17A	120.1
C3—C4—H4B	109.8	C18—C17—H17A	120.1
C5—C4—H4B	109.8	C13—C18—C17	120.34 (18)
H4A—C4—H4B	108.3	C13—C18—C19	120.35 (16)
C6—C5—C4	109.0 (2)	C17—C18—C19	119.28 (17)
C6—C5—C10	109.99 (18)	O2—C19—O1	117.32 (18)
C4—C5—C10	110.25 (19)	O2—C19—C18	124.05 (18)
C6—C5—H5A	109.2	O1—C19—C18	118.61 (16)
C4—C5—H5A	109.2	C15—C20—H20A	109.5
C10—C5—H5A	109.2	C15—C20—H20B	109.5
C5—C6—C7	109.59 (18)	H20A—C20—H20B	109.5
C5—C6—H6A	109.8	C15—C20—H20C	109.5
C7—C6—H6A	109.8	H20A—C20—H20C	109.5
C5—C6—H6B	109.8	H20B—C20—H20C	109.5
C7—C6—H6B	109.8	C22—C21—C11	117.76 (16)
H6A—C6—H6B	108.2	C22—C21—H21A	107.9
C9—C7—C6	109.28 (18)	C11—C21—H21A	107.9
C9—C7—C8	109.76 (17)	C22—C21—H21B	107.9
C6—C7—C8	109.59 (17)	C11—C21—H21B	107.9
C9—C7—H7A	109.4	H21A—C21—H21B	107.2
C6—C7—H7A	109.4	C23—C22—C27	118.57 (18)
C8—C7—H7A	109.4	C23—C22—C21	120.35 (19)
C7—C8—C1	111.10 (16)	C27—C22—C21	121.08 (18)
C7—C8—H8A	109.4	C22—C23—C24	121.7 (2)
C1—C8—H8A	109.4	C22—C23—H23A	119.2
C7—C8—H8B	109.4	C24—C23—H23A	119.2
C1—C8—H8B	109.4	C25—C24—C23	118.4 (2)
H8A—C8—H8B	108.0	C25—C24—C28	121.1 (2)
C3—C9—C7	108.92 (18)	C23—C24—C28	120.5 (2)
C3—C9—H9A	109.9	C26—C25—C24	120.80 (19)
C7—C9—H9A	109.9	C26—C25—H25A	119.6
C3—C9—H9B	109.9	C24—C25—H25A	119.6
C7—C9—H9B	109.9	C25—C26—C27	119.9 (2)
H9A—C9—H9B	108.3	C25—C26—H26A	120.1
C5—C10—C1	110.29 (17)	C27—C26—H26A	120.1
C5—C10—H10A	109.6	C26—C27—C22	120.7 (2)
C1—C10—H10A	109.6	C26—C27—H27A	119.7
C5—C10—H10B	109.6	C22—C27—H27A	119.7
C1—C10—H10B	109.6	C24—C28—H28A	109.5
H10A—C10—H10B	108.1	C24—C28—H28B	109.5
O1—C11—C12	109.21 (15)	H28A—C28—H28B	109.5
O1—C11—C21	109.47 (15)	C24—C28—H28C	109.5
C12—C11—C21	111.07 (16)	H28A—C28—H28C	109.5
O1—C11—C1	103.53 (14)	H28B—C28—H28C	109.5
			
C10—C1—C2—C3	−58.3 (2)	O1—C11—C12—C13	−48.1 (2)
C8—C1—C2—C3	58.0 (2)	C21—C11—C12—C13	72.7 (2)
C11—C1—C2—C3	179.39 (18)	C1—C11—C12—C13	−162.74 (15)
C1—C2—C3—C4	60.2 (2)	C11—C12—C13—C14	−148.46 (17)
C1—C2—C3—C9	−60.2 (3)	C11—C12—C13—C18	32.4 (2)
C9—C3—C4—C5	60.5 (2)	C18—C13—C14—C15	−0.3 (3)
C2—C3—C4—C5	−60.0 (2)	C12—C13—C14—C15	−179.42 (17)
C3—C4—C5—C6	−60.2 (2)	C13—C14—C15—C16	−0.3 (3)
C3—C4—C5—C10	60.6 (2)	C13—C14—C15—C20	179.94 (18)
C4—C5—C6—C7	60.6 (2)	C14—C15—C16—C17	0.4 (3)
C10—C5—C6—C7	−60.4 (2)	C20—C15—C16—C17	−179.86 (18)
C5—C6—C7—C9	−60.8 (2)	C15—C16—C17—C18	0.1 (3)
C5—C6—C7—C8	59.5 (2)	C14—C13—C18—C17	0.8 (3)
C9—C7—C8—C1	60.9 (2)	C12—C13—C18—C17	179.98 (17)
C6—C7—C8—C1	−59.1 (2)	C14—C13—C18—C19	178.59 (16)
C10—C1—C8—C7	57.8 (2)	C12—C13—C18—C19	−2.3 (3)
C2—C1—C8—C7	−58.3 (2)	C16—C17—C18—C13	−0.8 (3)
C11—C1—C8—C7	179.39 (16)	C16—C17—C18—C19	−178.54 (17)
C4—C3—C9—C7	−60.3 (2)	C11—O1—C19—O2	171.77 (17)
C2—C3—C9—C7	59.7 (2)	C11—O1—C19—C18	−9.5 (3)
C6—C7—C9—C3	60.1 (2)	C13—C18—C19—O2	167.88 (19)
C8—C7—C9—C3	−60.1 (2)	C17—C18—C19—O2	−14.3 (3)
C6—C5—C10—C1	60.3 (2)	C13—C18—C19—O1	−10.8 (3)
C4—C5—C10—C1	−60.0 (2)	C17—C18—C19—O1	166.98 (17)
C2—C1—C10—C5	57.5 (2)	O1—C11—C21—C22	50.9 (2)
C8—C1—C10—C5	−57.9 (2)	C12—C11—C21—C22	−69.7 (2)
C11—C1—C10—C5	−179.40 (17)	C1—C11—C21—C22	164.23 (17)
C19—O1—C11—C12	38.5 (2)	C11—C21—C22—C23	98.7 (2)
C19—O1—C11—C21	−83.3 (2)	C11—C21—C22—C27	−82.1 (2)
C19—O1—C11—C1	159.11 (15)	C27—C22—C23—C24	2.1 (3)
C10—C1—C11—O1	172.14 (15)	C21—C22—C23—C24	−178.67 (18)
C2—C1—C11—O1	−67.16 (19)	C22—C23—C24—C25	−0.8 (3)
C8—C1—C11—O1	52.08 (19)	C22—C23—C24—C28	178.27 (19)
C10—C1—C11—C12	−69.8 (2)	C23—C24—C25—C26	−1.0 (3)
C2—C1—C11—C12	50.9 (2)	C28—C24—C25—C26	179.9 (2)
C8—C1—C11—C12	170.09 (15)	C24—C25—C26—C27	1.5 (3)
C10—C1—C11—C21	55.1 (2)	C25—C26—C27—C22	−0.2 (3)
C2—C1—C11—C21	175.81 (17)	C23—C22—C27—C26	−1.5 (3)
C8—C1—C11—C21	−64.9 (2)	C21—C22—C27—C26	179.21 (19)

### Hydrogen-bond geometry (Å, °)

**Table:**

*D*—H···*A*	*D*—H	H···*A*	*D*···*A*	*D*—H···*A*
C23—H23A···O2^i^	0.95	2.65	3.548 (3)	157
C12—H12B···O2^i^	0.99	2.28	3.206 (2)	156

Symmetry codes: (i) *x*, *y*−1, *z*.

## References

[bb1] Bianchi, D. A., Blanco, N. E., Carrillo, N. & Kaufnam, T. S. (2004). *J. Agric. Food Chem.* **52**, 1923–1927. 10.1021/jf0351117 15053530

[bb2] Buntin, K., Rachid, S., Scharfe, M., Blöcker, H., Weissman, K. J. & Müller, R. (2008). *Angew. Chem. Int. Ed.* **47**, 4595–4599. 10.1002/anie.200705569 18461592

[bb3] Cremer, D. & Pople, J. A. (1975). *J. Am. Chem. Soc.* **97**, 1354–1358.

[bb4] Farrugia, L. J. (1997). *J. Appl. Cryst.* **30**, 565.

[bb5] Macrae, C. F., Bruno, I. J., Chisholm, J. A., Edgington, P. R., McCabe, P., Pidcock, E., Rodriguez-Monge, L., Taylor, R., van de Streek, J. & Wood, P. A. (2008). *J. Appl. Cryst.* **41**, 466–470.

[bb6] Oxford Diffraction (2006). *CrysAlis RED* and *CrysAlis CCD* Oxford Diffraction Ltd, Abingdon, England.

[bb7] Sheldrick, G. M. (2008). *Acta Cryst.* A**64**, 112–122. 10.1107/S0108767307043930 18156677

[bb8] Vícha, R., Nečas, M. & Potáček, M. (2006). *Collect. Czech. Chem. Commun.* **71**, 709–722.

